# Predicting Venous Thromboembolic Events in Patients with Coronavirus Disease 2019 Requiring Hospitalization: an Observational Retrospective Study by the COVIDIC Initiative in a Swiss University Hospital

**DOI:** 10.1155/2020/9126148

**Published:** 2020-11-06

**Authors:** Eleftheria Kampouri, Paraskevas Filippidis, Benjamin Viala, Marie Méan, Olivier Pantet, Florian Desgranges, Jonathan Tschopp, Jean Regina, Eleftherios Karachalias, Christophe Bianchi, Maxime G. Zermatten, Katia Jaton, Salah Dine Qanadli, Pierre-Alexandre Bart, Jean-Luc Pagani, Benoit Guery, Lorenzo Alberio, Matthaios Papadimitriou-Olivgeris, RegCOVID Research Group

**Affiliations:** ^1^Service of Infectious Diseases, Lausanne University Hospital (CHUV) and University of Lausanne (UNIL), Lausanne, Switzerland; ^2^Division of Internal Medicine, Lausanne University Hospital (CHUV) and University of Lausanne (UNIL), Lausanne, Switzerland; ^3^Service of Intensive Care, Lausanne University Hospital (CHUV) and University of Lausanne (UNIL), Lausanne, Switzerland; ^4^Starling Bank Limited, London, UK; ^5^Service of Hematology and Central Hematology Laboratory, Lausanne University Hospital (CHUV) and University of Lausanne (UNIL), Lausanne, Switzerland; ^6^Institute of Microbiology, Lausanne University Hospital (CHUV) and University of Lausanne (UNIL), Switzerland; ^7^Cardiothoracic and Vascular Division, Department of Diagnostic and Interventional Radiology, Lausanne University Hospital (CHUV) and University of Lausanne (UNIL), Lausanne, Switzerland; ^8^Service of Hospital Preventive Medicine, Lausanne University Hospital (CHUV) and University of Lausanne (UNIL), Lausanne, Switzerland

## Abstract

**Background:**

Coronavirus disease 2019 (COVID-19) can result in profound changes in blood coagulation. The aim of the study was to determine the incidence and predictors of venous thromboembolic events (VTE) among patients with COVID-19 requiring hospital admission. *Subjects and Methods*. We performed a retrospective study at the Lausanne University Hospital with patients admitted because of COVID-19 from February 28 to April 30, 2020.

**Results:**

Among 443 patients with COVID-19, VTE was diagnosed in 41 patients (9.3%; 27 pulmonary embolisms, 12 deep vein thrombosis, one pulmonary embolism and deep vein thrombosis, one portal vein thrombosis). VTE was diagnosed already upon admission in 14 (34.1%) patients and 27 (65.9%) during hospital stay (18 in ICU and nine in wards outside the ICU). Multivariate analysis revealed D-dimer value > 3,120 ng/ml (*P* < 0.001; OR 15.8, 95% CI 4.7-52.9) and duration of 8 days or more from COVID-19 symptoms onset to presentation (*P* 0.020; OR 4.8, 95% CI 1.3-18.3) to be independently associated with VTE upon admission. D-dimer value ≥ 3,000 ng/l combined with a Wells score for PE ≥ 2 was highly specific (sensitivity 57.1%, specificity 91.6%) in detecting VTE upon admission. Development of VTE during hospitalization was independently associated with D-dimer value > 5,611 ng/ml (*P* < 0.001; OR 6.3, 95% CI 2.4-16.2) and mechanical ventilation (*P* < 0.001; OR 5.9, 95% CI 2.3-15.1).

**Conclusions:**

VTE seems to be a common COVID-19 complication upon admission and during hospitalization, especially in ICU. The combination of Wells ≥ 2 score and D − dimer ≥ 3,000 ng/l is a good predictor of VTE at admission.

## 1. Introduction

Coronavirus disease 2019 (COVID-19) refers to the infection caused by the Severe Acute Respiratory Syndrome Coronavirus 2 (SARS-CoV-2), which was first reported in December 2019 in Wuhan, China, and subsequently spread worldwide rapidly gaining pandemic proportions, causing a large spectrum of manifestations varying from asymptomatic disease and mild respiratory symptoms to severe infection and death [1–3]. Profound changes in blood coagulation of these patients have already been described [4–6], and elevated D-dimer values upon admission have been recognized as a predictor of mortality [7, 8]. Even though the exact nature of the association between elevated D-dimer values and worse outcomes is not yet fully elucidated, venous thromboembolic events (VTE) could contribute to the higher morbidity and mortality of these patients.

Data on the association between SARS-CoV-2 and VTE are continuously emerging. Several studies have described the high incidence of VTE during severe SARS-CoV-2 infection in patients hospitalized in the Intensive Care Unit (ICU) [9–13], but data regarding the earlier stages of the disease or patients with less severe disease are still limited [11, 14]. In addition, it remains uncertain whether the SARS-CoV-2 infection is independently associated with an increased risk of VTE.

Activation of platelets and coagulation pathways are likely induced by the massive release of proinflammatory mediators reflected by the highly elevated inflammatory biomarkers during the course of the SARS-CoV-2 infection. The particular role of cytokines, such as TNF-*α*, IL-6, and the inhibitory cytokine IL-10, in association with a quantitative and functional lymphocyte dysregulation, for COVID-19 severity has been postulated by some studies [15, 16]. These features along with the loss of the normal antithrombotic and anti-inflammatory functions of endothelial cells and the leukocyte recruitment in the microvasculature, commonly described as thromboinflammation, seem to participate in the pathogenesis of thrombosis during COVID-19, in a way similar to sepsis [17, 18].

Our study is aimed at providing a thorough descriptive analysis of VTE occurring in distinct groups of patients according to the timing of presentation (upon admission, during hospitalization), in order to determine the VTE predictors and to evaluate the diagnostic performance of clinical scores and biomarkers. We also aim to assess the impact of the early VTE identification and the intensification of preventive anticoagulation strategies.

## 2. Subjects and Methods

This retrospective study was conducted at Lausanne University Hospital, a 1,500-bed tertiary care hospital and one of the five medical teaching hospitals in Switzerland. Adult patients with microbiologically documented SARS-CoV-2 infection admitted from February 28 to April 30, 2020, were included with a follow-up until May 5, 2020. SARS-CoV-2 infection was proven by real-time PCR, as previously described [19]. This work was performed as part of the COVID-19 Interdisciplinary Collaboration (the COVIDIC Initiative). The study was approved by the ethics committee of the Canton of Vaud (CER-VD 2020-00815) that exceptionally waived the need for informed consent allowing the inclusion of all hospitalized COVID-19 patients except those who refused the use of their clinical and laboratory data.

Patients' electronic health records were reviewed by infectious diseases residents to collect epidemiological (age, sex, comorbidities), clinical (symptoms, signs), laboratory (D-dimer, white blood cells, platelets, C-reactive protein, procalcitonin), prognostic and diagnostic scores (Padua, Revised Geneva score, simplified Geneva risk assessment model, Wells criteria), and radiological data. Data regarding known risk factors for VTE were collected, such as overt active cancer, current pregnancy, thrombophilia (hereditary or acquired), previous VTE, or a temporary predisposing factor in the previous month including paralysis, paresis, plaster immobilization of the lower limb, and major surgery [20–22]. All the data were entered in the Lausanne University Hospital's electronic database “regCOVID” using the REDCap® platform (Research Electronic Data Capture v8.5.24, Vanderbilt University, Tennessee, USA) [23].

The primary outcome was defined as VTE occurrence. VTE included pulmonary embolism (PE), deep vein thrombosis of the limbs (DVT), or thrombosis at other sites, including catheter-related thrombosis, confirmed by contrast-enhanced computed tomography (ceCT) pulmonary angiogram or Doppler-echography. No universal screening for asymptomatic VTE was performed. Hospital-acquired SARS-CoV-2 infection was defined as the initiation of symptoms five days after hospital admission. VTE was analyzed in two separate groups: upon admission and during hospitalization, the latter being also referred to in the literature as hospital-acquired thrombosis [24]. Based on prior studies showing that D-dimer could identify patients with poor prognosis at an early stage of COVID-19, the D-dimer measurement was initially proposed by the institutional guidelines in all patients admitted with respiratory insufficiency [8].

Data on anticoagulation therapy were collected including the type of molecule: low molecular weight heparin (LMWH), unfractionated heparin (UFH) and oral anticoagulants (both direct oral anticoagulants and vitamin K antagonists), dosage, route, and duration of administration. A patient was considered to be on anticoagulation treatment in case of administration of any type of anticoagulation in prophylactic or therapeutic dosage, for more than 72 h before VTE diagnosis. Intermediate-dosage thromboprophylaxis protocol (for creatinineclearance ≥ 30 ml/minUFH sc 5,000 UI tid or enoxaparin 40 mg bid (<120 kg) or 60 mg bid (≥120 kg); for creatinine clearance <30 ml/min UFH iv 200 UI/kg/24 h) was included on April 6, 2020 in the internal recommendations on thromboprophylaxis in COVID-19 patients admitted in the ICU [25]. Because the level of awareness of VTE complications related to COVID-19 rose rapidly during the pandemic period as a consequence of the accumulation of reports in the literature, the study period was divided into three periods: the first period from February 28 to March 25 (low awareness of SARS-CoV-2 thrombogenic potential), second from March 26 to April 5 (increased level of awareness), and third from April 6 to May 5 (change in prophylactic anticoagulation in ICU patients).

Data analyses were performed using the open-source programming language Python (Python Software Foundation, Wilmington, DE, USA) and the associated libraries for statistical analysis such as SciPy and StatsModels. Fisher exact test or chi-square was used for categorical variables, whereas Mann–Whitney *U* test was used for numerical ones. In case of missing data, the univariate analysis included only patients with available data. Receiver operating characteristic (ROC) curves for VTE development upon admission and hospitalization were generated for D-dimer, and the optimal cutoff value was calculated with Youden's index (Supplementary Figure 1). Logistic regression was used to determine the risk factors for VTE development. Odds ratios (ORs) and 95% confidence intervals (CIs) were calculated to evaluate the strength of any association. All statistical tests were 2-tailed, and *P* < 0.05 was considered statistically significant.

## 3. Results

**Figure 1:**
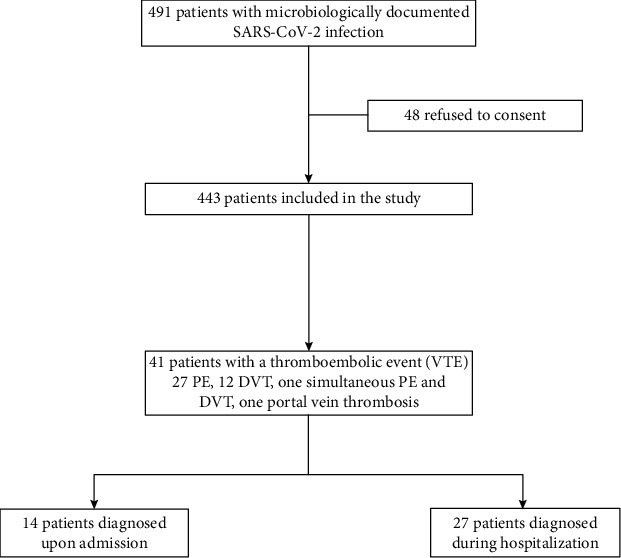
Flowchart of included patients.

During the study period, 491 patients with microbiologically documented SARS-CoV-2 infection were admitted to the hospital. Among them, 48 patients had previously refused to consent to the use of their clinical data and were excluded. Thus, a total of 443 patients hospitalized with SARS-CoV-2 infection were included in this study (Figure 1).

Overall, a thromboembolic event was identified in 41 patients (9.3%); 27 patients had PE (four saddle, 10 lobar, 13 segmental or subsegmental), 12 had DVT (seven proximal and five distal; seven occurring in the lower extremities, two in the upper extremities, three in the jugular vein), one patient had simultaneously lobar PE and distal DVT of the lower limb, and one had portal vein thrombosis associated with a concomitant episode of acute cholecystitis. All VTE patients had a D-dimer value measurement the same day of diagnosis or the day before. Fourteen patients (34.1%) were diagnosed upon admission (three admitted directly at ICU and 11 at wards), from whom 11 (78.6%) had PE. Regarding the 27 patients (65.9%) with VTE during hospitalization, VTE was diagnosed at a median of eight days from admission; from these patients, nine were hospitalized in medical wards and 18 in the ICU. Supplementary Table 1 provides a descriptive analysis of the characteristics of all patients with VTE.

**Figure 2:**
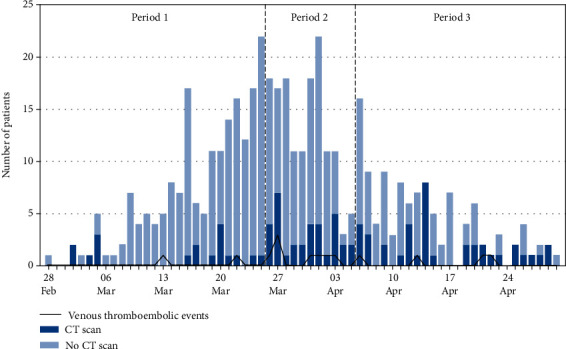
Daily number of COVID-19 patients admitted and those with confirmed VTE and CT scan performed upon admission.

**Figure 3:**
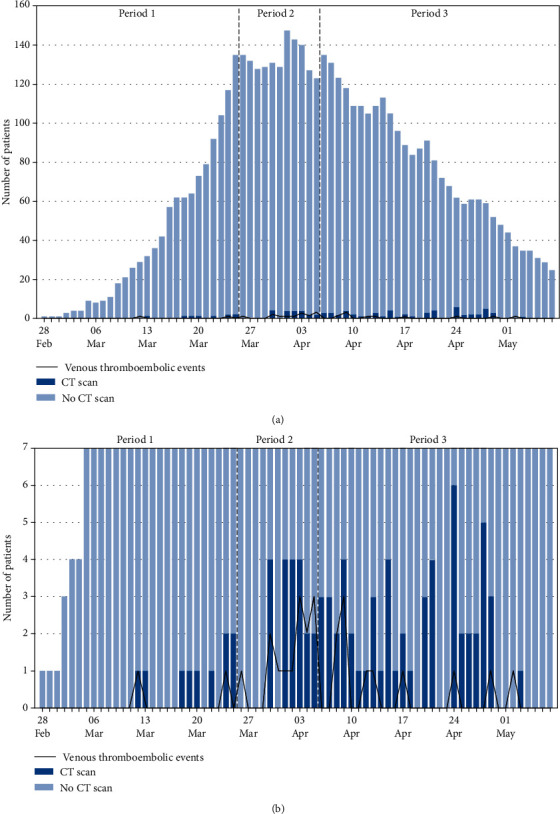
(a), (b) Daily number of COVID-19 patients being hospitalized and those with confirmed VTE and CT scan performed during hospitalization.

A total of 171 chest ceCT scans were performed in 135 patients (30.5%) during the study period.  Figure 2 illustrates the daily number of patients who had a CT scan performed upon admission and the number of VTE diagnoses out of the total number of patients admitted on the same day. Figures 3(a) and 3(b) show the daily proportion of patients with a CT scan performed during hospitalization out of the total number of patients present in the hospital and the number of VTE diagnoses on the same day. Upon admission, we observed an increase in performed ceCT scans (*P* < 0.001) and VTE diagnoses per 100 admissions (*P* 0.020) during the second period as compared to the first; no difference in ceCT scans (*P* 0.464) or VTE diagnoses (*P* 1.000) was observed between the second and third period. During hospitalization, ceCT scans increased steadily throughout the three periods. However, while VTE diagnoses per 1,000-patient-days increased from the first to second period (*P* 0.041), a tendency to decrease was observed from the second to the third one (*P* 0.056) (Table 1).

Patients' characteristics and univariate analysis of predictors of VTE upon admission are shown in Table 2. D-dimer values were available for 363 patients (81.9%). Multivariate analysis revealed D-dimer value > 3,120 ng/ml (*P* < 0.001; OR 15.8, 95% CI 4.7-52.9) and duration of 8 days or more from COVID-19 symptoms onset to presentation (*P* 0.020; OR 4.8, 95% CI 1.3-18.3) to be independently associated with VTE upon admission.  Table 3 depicts the patients' characteristics and univariate analysis of predictors of VTE during hospitalization. D-dimer values were available for 373 (86.9%). D-dimer value > 5,611 ng/ml (*P* < 0.001; OR 6.3, 95% CI 2.4-16.2) and mechanical ventilation (*P* < 0.001; OR 5.9, 95% CI 2.3-15.1) were independent predictors for VTE during hospitalization. The abovementioned D-dimer values were the optimal cutoffs defined by ROC-curve analysis (Supplementary Figure 1).

**Figure 4:**
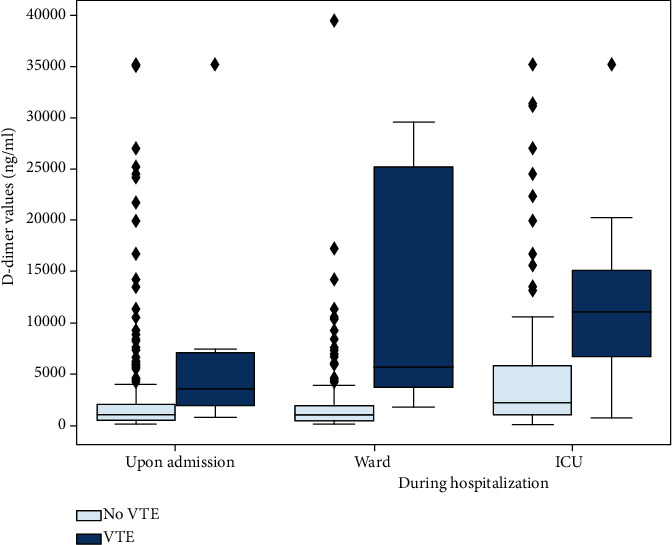
(a) D-dimer values (ng/ml) in patients in the emergency room for the prediction of VTE upon admission (*P* < 0.001). (b) D-dimer values (ng/ml) for the prediction of VTE during hospitalization in wards (*P* < 0.001). (c) D-dimer values (ng/ml) for the prediction of VTE during hospitalization in ICU (*P* < 0.001). For (b) and (c), the peak D-dimer value during their stay was used for patients without VTE and the last value before the diagnosis of the thrombotic event for VTE patients.

D-dimer values were available for 363 patients (81.9%) upon admission and for 373 (86.9%) during hospitalization.  Table 4 shows the performance of different combinations of D-dimer values and Wells score for PE in predicting VTE upon admission. The presence of either a Wells score forPE ≥ 2points or a D-dimervalue ≥ 1,000 ng/mlis the most sensitive for PE diagnosis (sensitivity 92.9%, specificity 46.9%). On the other hand, a D-dimer value ≥ 3,000 ng/l combined with a Wells score for PE ≥ 2 was associated with the highest specificity (sensitivity 57.1%, specificity 91.6%, accuracy 0.905), while D-dimer value ≥ 3,000 ng/l alone was less specific for this diagnosis (sensitivity 71.4%, specificity 87.9%, accuracy 0.797).  Figure 4 (patients in the emergency room) shows the D-dimer values for the prediction of VTE upon admission (*P* < 0.001), while Figures 4(b) (during hospitalization in wards, *P* < 0.001) and (c) (during hospitalization in the ICU, *P* < 0.001) depict the peak D-dimer value for non-VTE and the last value before the diagnosis of the thrombotic event for VTE patients.

## 4. Discussion

In this study, the rate of VTE among hospitalized patients with COVID-19 was 9.3% which is similar to that reported previously among COVID-19 inpatients [14, 26] and much higher than the VTE incidence previously described among inpatients with influenza and influenza-associated pneumonia (1.0-3.4%) [27, 28], while no published data are available concerning the VTE prevalence in previous coronavirus infections SARS-CoV-1 and MERS-CoV.

Of particular note, a large proportion of VTE was diagnosed upon admission (14 out of 443 patients; 3.2%). To the best of our knowledge, this is the first study to find an association between a prolonged duration of COVID-19-related symptoms and VTE diagnosis upon admission. This observation could be explained by a greater degree of immobilization (possibly due to infection control measures) and/or a longer exposure to the systemic inflammatory response [29], contributing to an increased risk of VTE already present before admission. As previously shown, the peak of systemic inflammatory response reflected on the greatest severity of lung lesions (ground-glass opacities, consolidations) was found 9-13 days from the onset of the initial symptoms [29]. This observation should prompt evaluation of thromboprophylaxis for some patients in the outpatient setting. Similarly, the risk of thrombosis after discharge still needs to be assessed, as it may persist for several weeks after acute infection by analogy with other respiratory infections [30], requiring evaluation of a prolonged duration of thromboprophylaxis after discharge.

In our study, ceCT scans and other diagnostic testing were performed as part of standard care, according to physicians' discretion; 30.5% of patients had at least one ceCT scan performed, the percentage comparable to previous reports [14, 31]. During the study period, an interesting pattern was observed with three distinct periods. ceCT scans were less frequently performed during the initial period associated with fewer VTE diagnoses. This finding could reflect a lower level of awareness of the risk of thromboembolic complications in the setting of COVID-19. Chest ceCT scans were more frequently performed during the second period, with a subsequent rise in VTE diagnoses (both upon admission and during hospitalization). Finally, in the third period, despite the high number of ceCT scans performed, a trend towards fewer VTE diagnoses was observed during hospitalization. This pattern could reflect the effect of more aggressive anticoagulation strategies implemented in ICU-hospitalized patients on April 6, 2020 [25].

Hospitalization in the ICU and especially mechanical ventilation were associated with VTE occurrence. The high rate in the ICU setting (22.3%; 21 out of 94 patients) is consistent with the current bibliography, but seems lower than most of the previous studies, especially compared to a recent meta-analysis showing an overall rate of 30.4% among ICU patients [12, 13, 26]. Interestingly, Helms et al. reported a higher rate in patients with COVID-19-related acute respiratory distress syndrome (ARDS) in comparison with patients with ARDS of other causes [9]. The importance of these clinical incidence rates is further highlighted by a recent series of 12 consecutive autopsy reports showing the presence of VTE in 58% of cases, who went unrecognized before death [32]. Among patients hospitalized in wards, the incidence of VTE (5.9%; 20 out of 335 patients) was also inferior to the one reported in the abovementioned meta-analysis (13%) [25, 26]. These lower rates in our study could be explained by the intensification of prophylactic anticoagulation during the study period, as well as the earlier hospitalization of many patients presenting less severe complications compared to other countries with a more significant healthcare overload [14].

While an isolated D-dimer value alone cannot reliably be used to assess the indication for ceCT scan, it helps to establish useful strategies for PE diagnosis. According to our data, PE was less likely upon admission in case of a Wells score of ≤2 points and a D-dimer value of ≤1,000 ng/ml, and ceCT scan may therefore not be required. Moreover, when diagnostic imaging for PE is not possible, empiric therapeutic anticoagulation should be considered if the Wells score is ≥2 points and the D-dimer value is ≥3,000 ng/l. The D-dimer values upon admission and during hospitalization (ward, ICU) showed a significant difference among patients with VTE and those without (Figure 4). Defining a more accurate D-dimers' threshold could be an interesting diagnostic strategy, but requires further prospective evaluation.

Several limitations of our work are worth noting. First, this is a retrospective, single-center study; thus, the results may not be safely generalized. Second, suspicion and diagnosis of VTE were at clinicians' discretion, and no active surveillance was systematically performed during the study period. Therefore, the incidence of VTE was probably underestimated, as suggested by the abovementioned autopsy reports [32]. Third, the VTE definition differs from classical VTE studies, which exclude VTE occurring at other sites than the lower limbs and pulmonary embolism. However, all previous studies in COVID-19 setting included VTE of all localisations [9, 11, 12, 13, 14]. Finally, we provided a follow-up only for the duration of the hospitalization, whereas a longer follow-up of at least three months from infection onset may be desirable.

## 5. Conclusion

VTE seems to be a common complication of SARS-CoV-2 infection already upon admission and during hospitalization, especially in ICU. Time from symptoms' onset to admission seems to be a major clinical risk factor for patients diagnosed for VTE upon admission. Our study provides for the first time a 3-phase timeline illustration of the impact of increased awareness for VTE and the beneficial role of a subsequent intensification of the preventive anticoagulation strategies in patients requiring hospitalization because of COVID-19. The combination of Wells' score with the D-dimer value at admission can be a useful tool to guide empiric anticoagulation therapy when diagnostic imaging is not possible or available. Furthermore, based on the high rate of early VTE present upon admission, there appears to be an urgent need to study the potential benefit of thromboprophylaxis for patients with SARS-CoV-2 infection in the ambulatory setting.

## Figures and Tables

**Table 1:** CT-scan and VTE per 100 admissions or per 1,000-patient-days in the three periods.

	Period 1 February 28 to March 25	Period 2 March 26 to April 5	Period 3 April 6 to May 7	*P* ^a^	*P* ^b^
VTE upon admission					
ceCT scan performed	18	34	31	<0.001	0.464
VTE	2	8	4	0.020	1.000
Admissions	185	145	113	-	-
ceCT scan per 100 admissions	9.7	23.4	27.4	-	-
VTE per 100 admissions	1.1	5.5	3.5	-	-
VTE during hospitalization					
ceCT-scan performed	10	20	56	0.328	0.041
VTE	2	14	11	0.016	0.056
Patient days	1111	1528	2543	-	-
ceCT scan per 1,000-patient-days	9.0	13.1	22.0	-	-
VTE per 1,000-patient-days	1.8	9.2	4.3	-	-

ceCT: contrast-enhanced computed tomography; VTE: venous thromboembolism. ^a^Comparison of periods 1 and 2. ^b^Comparison of periods 2 and 3.

**Table 2:** Patients' characteristics and univariate analysis of factors associated with VTE upon admission.

Characteristics	Without VTE (*n* = 429)	VTE (*n* = 14)	*P*
Demographics			
Age (years)	69.0 (55.0-81.0)	59.0 (49.5-70.0)	0.051
Male gender	246 (57.3%)	10 (71.4%)	0.412
Comorbidities			
Diabetes mellitus	105 (24.5%)	4 (28.6%)	0.754
Chronic obstructive pulmonary disease	33 (7.7%)	1 (7.1%)	1.000
Chronic heart failure	38 (8.9%)	1 (7.1%)	1.000
Chronic kidney disease	65 (15.2%)	3 (21.4%)	0.460
Cirrhosis	16 (3.7%)	0 (0.0%)	1.000
Malignancy (hematological or solid organ)	51 (11.9%)	0 (0.0%)	0.386
Previous VTE	34 (7.9%)	3 (21.4%)	0.103
Obesity	104 (24.2%)	4 (28.6%)	0.753
Charlson comorbidity index	5.0 (2.0-6.0)	2.0 (1.0-4.0)	0.077
Symptoms^a^			
Community-acquired infection	362 (84.4%)	13 (92.9%)	0.705
Days from symptoms onset	7.0 (3.0-10.0)	10.0 (8.2-12.2)	0.002
Duration of symptoms ≥8 days	162 (37.8%)	11 (78.6%)	0.004^b^
Fever	277 (64.6%)	11 (78.6%)	0.396
Cough	280 (65.3%)	10 (71.4%)	0.779
Dyspnea	234 (54.5%)	12 (85.7%)	0.027
Thoracic pain	49 (11.4%)	5 (35.7%)	0.019
Signs^a^			
Temperature (°C)	38.1 (37.3-38.7)	37.9 (37.0-38.5)	0.301
Systolic blood pressure (mmHg)	117.0 (102.2-127.0)	107.0 (100.0-130.0)	0.590
Heart rate (beats/min)	92.0 (82.0-104.0)	103.0 (87.0-111.0)	0.177
Respiratory rate (breath/min)	25.0 (21.0-31.0)	26.0 (24.0-29.0)	0.805
Glasgow Coma Scale	15.0 (15.0-15.0)	15.0 (15.0-15.0)	0.212
Laboratory findings^a^			
White blood cells (G/l)	6.1 (4.7-8.8)	10.0 (5.9-13.2)	0.014
D-dimer (ng/ml) (among 363 patients)	1039.0 (549.0-2020.0)	3610.0 (1934.0-7093.8)	<0.001
D-dimer >3,120 ng/ml	52 (12.1%)	10 (71.4%)	<0.001^b^
C-reactive protein (mg/l) (among 413 patients)	61.5 (21.0-124.2)	91.0 (53.0-187.0)	0.225
Procalcitonin (ng/ml) (for 321 patients)	0.2 (0.1-0.4)	0.2 (0.1-0.5)	0.876
Radiological findings^a^			
Normal X-ray or ceCT scan	92 (21.4%)	3 (21.4%)	1.000
Bilateral infiltrates on X-ray or ceCT scan	247 (57.6%)	11 (78.6%)	0.168
Prior administration of therapeutic or prophylactic anticoagulation	88 (20.5%)	0 (0.0%)	0.083
Scores (prognostic or diagnostic)^a^			
Padua score	6.0 (5.0-7.0)	6.0 (5.0-6.0)	0.814
Revised Geneva score	5.0 (3.0-6.0)	5.0 (4.2-6.8)	0.205
Revised Geneva score ≥ 4	314 (73.2%)	12 (85.7%)	0.373
Simplified Geneva risk assessment model	5.0 (4.0-6.0)	5.0 (4.2-5.0)	0.809
Simplified Geneva risk assessment model ≥ 3	396 (92.3%)	12 (85.7%)	0.305
Wells criteria	1.0 (0.0-1.5)	3.8 (1.9-4.5)	<0.001
Wells criteria ≥ 2	97 (22.6%)	10 (71.4%)	<0.001
Outcome			
Type of discharge			
Discharge at home	217 (50.6%)	10 (71.4%)	
Transfer to other acute care hospital	48 (11.2%)	2 (14.3%)	
Rehabilitation	82 (19.1%)	0 (0.0%)	
Still hospitalized	23 (5.4%)	1 (7.1%)	
Death	59 (13.8%)	1 (7.1%)	0.704
Length of hospitalization	10.9 (4.0-13.0)	9.9 (3.0-13.0)	0.688

Data are number (%) of patients or median (*Q*_1_-*Q*_3_). ^a^Upon admission. ^b^Factors included in the multivariate analysis. ceCT: contrast-enhanced computed tomography.

**Table 3:** Patients' characteristics and univariate analysis of factors associated with VTE during hospitalization.

Characteristics	Without VTE (*n* = 402)	VTE (*n* = 27)	*P*
Days at risk^a^	8.0 (4.0-12.0)	8.0 (6.5-12.0)	0.319
Demographics			
Age (years)	70.0 (55.0-81.0)	62.0 (58.0-68.5)	0.039
Male gender	224 (55.7%)	22 (81.5%)	0.009^b^
Comorbidities			
Diabetes mellitus	99 (24.6%)	6 (22.2%)	1.000
Chronic obstructive pulmonary disease	29 (7.2%)	4 (14.8%)	0.144
Chronic heart failure	36 (9.0%)	2 (7.4%)	1.000
Chronic kidney disease	65 (16.2%)	0 (0.0%)	0.022
Cirrhosis	15 (3.7%)	1 (3.7%)	1.000
Malignancy (hematological or solid organ)	48 (11.9%)	3 (11.1%)	1.000
Previous VTE	33 (8.2%)	1 (3.7%)	0.712
Obesity	96 (23.9%)	8 (29.6%)	0.491
Charlson comorbidity index	5.0 (2.0-7.0)	3.0 (2.0-5.0)	0.025
Laboratory findings^c^			
White blood cells (G/l)	7.2 (5.4-10.2)	8.6 (7.4-9.7)	0.107
D-dimer (ng/ml) (among 373 patients)	1252.5 (629.8-2722.5)	10835.0 (4748.5-16679.0)	<0.001
D − dimer > 5611 ng/ml	48 (11.9%)	17 (63.0%)	<0.001^b^
C-reactive protein (mg/l) (among 411 patients)	74.5 (28.2-146.8)	51.0 (25.0-213.0)	0.630
Procalcitonin (ng/ml) (for 344 patients)	0.2 (0.1-0.5)	0.2 (0.1-0.7)	0.696
Radiological findings^c^			
Normal X-ray or ceCT scan	90 (22.4%)	0 (0.0%)	0.012
Bilateral infiltrates on X-ray or ceCT scan	226 (56.2%)	26 (96.3%)	**<**0.001
Complications/treatments^c^			
ICU admission	76 (18.9%)	18 (66.7%)	<0.001
Mechanical ventilation	55 (13.7%)	18 (66.7%)	<0.001^b^
Acute respiratory distress syndrome	76 (18.9%)	17 (63.0%)	<0.001
Prior anticoagulation^d^			
No anticoagulation	88 (21.9%)	8 (29.6%)	0.345
Prophylactic anticoagulation^e^	250 (62.2%)	17 (63.0%)	0.901
Therapeutic anticoagulation	72 (17.9%)	1 (3.7%)	0.064
Scores (prognostic or diagnostic)			
Padua score	6.0 (5.0-7.0)	5.0 (5.0-6.0)	0.350
Revised Geneva score	5.0 (3.0-6.0)	4.0 (3.0-6.5)	0.442
Revised Geneva score ≥ 4	299 (74.4%)	15 (55.6%)	0.056
Simplified Geneva risk assessment model	5.0 (4.0-6.0)	5.0 (4.5-5.0)	0.418
Simplified Geneva risk assessment model ≥ 3	370 (92.0%)	26 (96.3%)	0.710
Wells criteria	1.2 (0.0-1.5)	3.0 (3.0-3.5)	<0.001
Wells criteria ≥ 2	78 (19.4%)	21 (77.8%)	<0.001
Outcome			
Type of discharge			
Discharge at home	208 (51.7%)	9 (33.3%)	
Transfer to other acute care hospital	41 (10.2%)	7 (25.9%)	
Rehabilitation	78 (19.4%)	4 (14.8%)	
Still hospitalized	18 (4.5%)	5 (18.5%)	
Death	57 (14.2%)	2 (7.4%)	0.561
Length of hospitalization	9.9 (4.0-12.0)	27.9 (16.0-24.0)	<0.001

Data are number (%) of patients or median (*Q*_1_-*Q*_3_). ^a^From admission until VTE. ^b^Factors included in the multivariate analysis. ^c^Last value before VTE diagnosis for patients with VTE; peak value during hospitalization for patients without VTE. ^d^From admission until 72 h before VTE for VTE patients; from admission to discharge for patients without VTE. ^e^Characterized as a dose lesser to the therapeutic one. ceCT: contrast-enhanced computed tomography.

**Table 4:** Performance of different combinations of D-dimer values and Wells score for PE in predicting VTE upon admission.

	Sensitivity (%)	Specificity (%)	PPV (%)	NPV (%)	Accuracy
D-dimer value ≥ 3,000 ng/ml	71.4	87.9	99.0	87.4	0.797
Wells score for PE ≥ 2 points	71.4	77.4	9.3	98.8	0.772
Wells score for PE ≥ 2 points and D-dimer value ≥ 3,000 ng/ml	57.1	91.6	18.2	98.5	0.905
Wells score for PE ≥ 2 points or D-dimer value ≥1,000 ng/ml	92.9	46.9	5.4	99.5	0.483

PPV: positive predictive value; NPV: negative predictive value.

## Data Availability

The datasets generated during and/or analyzed during the current study are available from the corresponding author on reasonable request.

## Abbreviations

ARDS: Acute respiratory distress syndrome
ceCT: Contrast-enhanced computed tomography
CIs: Confidence intervals
COVID-19: Coronavirus disease 2019
DVT: Deep vein thrombosis of the limbs
ICU: Intensive care unit
LMWH: Low molecular weight heparin
ORs: Odds ratios
PE: Pulmonary embolism
SARS-CoV-2: Severe Acute Respiratory Syndrome Coronavirus 2
UFH: Unfractionated heparin
VTE: Venous thromboembolic events.

## Supplementary Materials

Supplementary Materials Supplementary Table 1: Descriptive analysis of patients with VTE upon admission or during hospitalization. Supplementary Figure 1: ROC curve of D-dimers in diagnosis of VTE among patients with VTE upon admission (1A), or during hospitalization (1B).
