# Supplementary material for: Predicting Venous Thromboembolic Events in Patients with Coronavirus Disease 2019 Requiring Hospitalization: an Observational Retrospective Study by the COVIDIC Initiative in a Swiss University Hospital
Source: Biomed Res Int. 2020 Nov 6;2020:9126148. doi: 10.1155/2020/9126148 (PMC7656238; doi:10.1155/2020/9126148)

**Supplementary Table 1:** Descriptive analysis of patients with VTE upon admission or during hospitalization

| Gender, Age (years) | VTE Type | BMI (kg/m2) | VTE Risk Factors^*^ or other relevant comorbidities | Days from symptom onset to VTE diagnosis | Days from admission to VTE diagnosis | Reason for imaging^†^ | D-dimers (ng/ml)^‡^ | Prior anticoagulation | Outcome^§^ |
| --- | --- | --- | --- | --- | --- | --- | --- | --- | --- |
| Upon admission | | | | | | | | | |
| M, 45 | PE | 21 | No | 10 | - | Clinical suspicion | 1,168 | No | Discharge |
| M, 34 | PE | 32 | **Obesity,** Dermatomyositis, NSIP | 9 | - | Clinical suspicion | 797 | No | Discharge |
| M, 83 | Proximal lower limb DVT | 23 | Hypertension, CKD | 15 | - | Clinical suspicion | >35,200 | No | Discharge |
| F, 28 | PE | 19 | **Combined contraceptive pill** | 10 | - | Clinical suspicion | 3,177 | No | Discharge |
| M, 64 | PE | 27 | Hypertension, Diabetes | 18 | - | D-dimers | 5,982 | No | Discharge |
| F, 67 | PE | 21 | **Prior VTE** | 7 | - | Unknown | NA | No | Transfer |
| M, 49 | PE | 36 | **Obesity,** diabetes | 16 | - | Clinical suspicion | 6,016 | No | Discharge |
| M, 71 | Proximal lower limb DVT | 25 | Diabetes | 8 | - | Clinical suspicion | NA | No | Death |
| M, 57 | PE | 24 | No | 10 | - | D-dimers | >35,200 | No | Discharge |
| F, 80 | Distal lower limb DVT | 21 | **Prior VTE**, COPD, CHF, CKD, Atrial Fibrillation | 5 | - | Clinical suspicion | 983 | No | Discharge |
| M, 72 | PE | 22 | **Lung carcinoma**  COPD, Atrial fibrillation | 4 | - | Clinical suspicion | 25,197 | Acenocoumarol  (INR ≥2) | Discharge |
| M, 72 | PE | 26 | Hypertension | 14 | - | Clinical suspicion | 3,376 | No | Discharge |
| M, 51 | PE | 30 | **Obesity, prior VTE** | 6 | - | D-dimers | >35,200 | No | Transfer |
| M, 61 | PE | 25 | No | 11 | - | New RBBB | 11,038 | No | Hospitalized |
| During hospitalization | | | | | | | | | |
| Patients Hospitalized in Intensive Care Unit upon diagnosis | | | | | | | | | |
| M, 67 | PE | 25 | No |  | 3 | D-dimers | 20,361 | No | Discharge |
| M, 70 | Jugular-vena cava DVT | 23 | CHF, Hypertension | - | 8 | Clinical suspicion | NA/>35,200 | Enoxaparin 40 mg bid | Hospitalized |
| M, 63 | PE | 26 | No | - | 14 | Clinical suspicion | 4,014/ 10,620 | UFH 10,000 UI/24h | Hospitalized |
| M, 65 | PE | 40 | **Obesity**, COPD, Hypertension,  Diabetes, Cirrhosis | - | 6 | Unknown | NA/NA | No | Transfer |
| F, 47 | Distal upper limb DVT | 35 | **Obesity** | - | 9 | Clinical suspicion | 6,270/1,864 | No (hemorrhage) | Hospitalized |
| F, 60 | PE | 27 | No | - | 8 | D-dimers | 1,022/20,179 | Enoxaparin 40 mg bid | Hospitalized |
| F, 60 | PE | 26 | Hypertension | - | 15 | Clinical suspicion | 366/1,915 | Enoxaparin 40 mg bid | Discharge |
| M, 69 | PE | 28 | No | - | 8 | Clinical suspicion | 1,452/10,846 | Enoxaparin 40 mg qd | Transfer |
| M, 57 | PE | 36 | **Obesity**, Hypertension | - | 11 | D-dimers | 14,878/22,021 | UFH 5,000 UI tid | Transfer |
| M, 62 | PE, distal lower limb DVT | 23 | No | - | 8 | Clinical suspicion | NA/NA | UFH prophylactic^#^ | Hospitalized |
| F, 46 | PE | 39 | **Obesity,** Hypertension | - | 14 | D-dimers | 1,119/10,958 | Enoxaparin 40 mg bid | Transfer |
| M, 62 | Distal lower limb DVT | 23 | Hypertension | - | 7 | D-dimers | 914/9,343 | UFH 10,000 UI/24h | Discharge |
|  | Proximal lower limb DVT |  |  |  | 16 | Clinical suspicion | 4,819/34,138 | UFH therapeutic |  |
| M, 56 | PE | 28 | No | - | 10 | D-dimers | 706/14,201 | Enoxaparin 40 mg qd  UFH 10,000 UI/24h^**^ | Discharge |
| M, 60 | Proximal lower limb DVT (CVC) | 25 | No | - | 13 | Clinical suspicion | 1,032/19,780 | No (thrombocytopenia) | Death |
| M, 71 | Distal lower limb DVT | 32 | **Obesity**, recent MI, Hypertension, Diabetes | - | 13 | Clinical suspicion | 2,225/10,733 | UFH 10,000 UI/24h | Death |
| M, 60 | Jugular-vena cava DVT | 25 | CHF, COPD | - | 12 | Clinical suspicion | 913/35,200 | Enoxaparin 40 mg qd, UFH 10,000 UI/24h^**^ | Transfer |
| M, 55 | PE | 35 | **Obesity**, Hypertension, Diabetes | - | 9 | Clinical suspicion | 2,786/11,366 | Enoxaparin 40 mg qd | Transfer |
| M, 68 | Jugular DVT (CVC) | 35 | **Obesity**, Hypertension, Diabetes | - | 10 | Clinical suspicion | 3,278/14,178 | UFH 5,000 UI tid | Transfer |
| Patients Hospitalized in General Ward upon VTE diagnosis | | | | | | | | | |
| M, 59 | PE | 23 | No |  | 2 | Clinical suspicion | 5,675 | No | Discharge |
| F, 57 | PE | 34 | **Obesity, Prior VTE** |  | 2 | Clinical suspicion | NA | No | Discharge |
| F, 53 | Distal lower limb DVT | 32 | **Obesity**, Diabetes, CKD |  | 3 | Clinical suspicion | 3,844 | No | Discharge |
| M, 80 | PE | 20 | **Multiple myeloma**  Rheumatoïd arthritis | - | 21 | Clinical suspicion | 1,267/2,942 | Enoxaparin 40 mg qd | Discharge |
| M, 66 | Portal vein thrombosis | 25 | No | - | 8 | Incidental finding | 3,822/NA | Enoxaparin 40 mg qd | Discharge |
| M, 27 | PE | 24 | No | - | 12 | Clinical suspicion | 412/NA | No | Discharge |
| M, 81 | Proximal upper limb DVT | 25 | COPD, Hypertension, Diabetes,  Polymyalgia rheumatica | - | 6 | Clinical suspicion | 35,043/NA | No | Discharge |
| M, 76 | PE | 20 | **Lymphoma**  (autologous HSCT) | - | 7 | Clinical suspicion | NA/NA | No | Discharge |
| M, 62 | PE | 23 | NSIP, Diabetes | - | 5 | D-dimers | 1,734/29,530 | No | Discharge |

BMI: body mass index, CHF: chronic heart failure, CKD: Chronic kidney disease, COPD: chronic obstructive pulmonary disease, CVC: central venous catheter, DVT: deep vein thrombosis, HSCT: hematopoietic stem cell transplantation, INR: international normalized ratio, ICU: intensive care unit, MI: myocardial infarction (recent: during the last 3 months), NA: not available, NSIP: nonspecific interstitial pneumonia, PE: pulmonary embolism, RBBB: right bundle branch block, UFH: unfractionated heparin, VTE: venous thromboembolic event

^*^VTE relevant risk factors appear in bold

^†^clinical suspicion was based on signs or symptoms compatible with VTE, deterioration of respiratory parameters without alternative explanation, lack of clinical improvement, hemodynamic compromise; unknown due to diagnosis in another hospital before patient transfer

^‡^for VTE upon admission, D-dimers upon diagnosis are shown; for VTE during hospitalization, D-dimers lowest value and value upon diagnosis are shown

^§^discharge at home or rehabilitation center

^¶^patients with hospital-acquired infection

^#^dosage not available (transfer from other hospital)

^**^prophylactic regimens not used simultaneously

**Supplementary Figure 1:** ROC curve of D-dimers in diagnosis of VTE among patients with VTE upon admission (**1A**), or during hospitalization (**1B**)


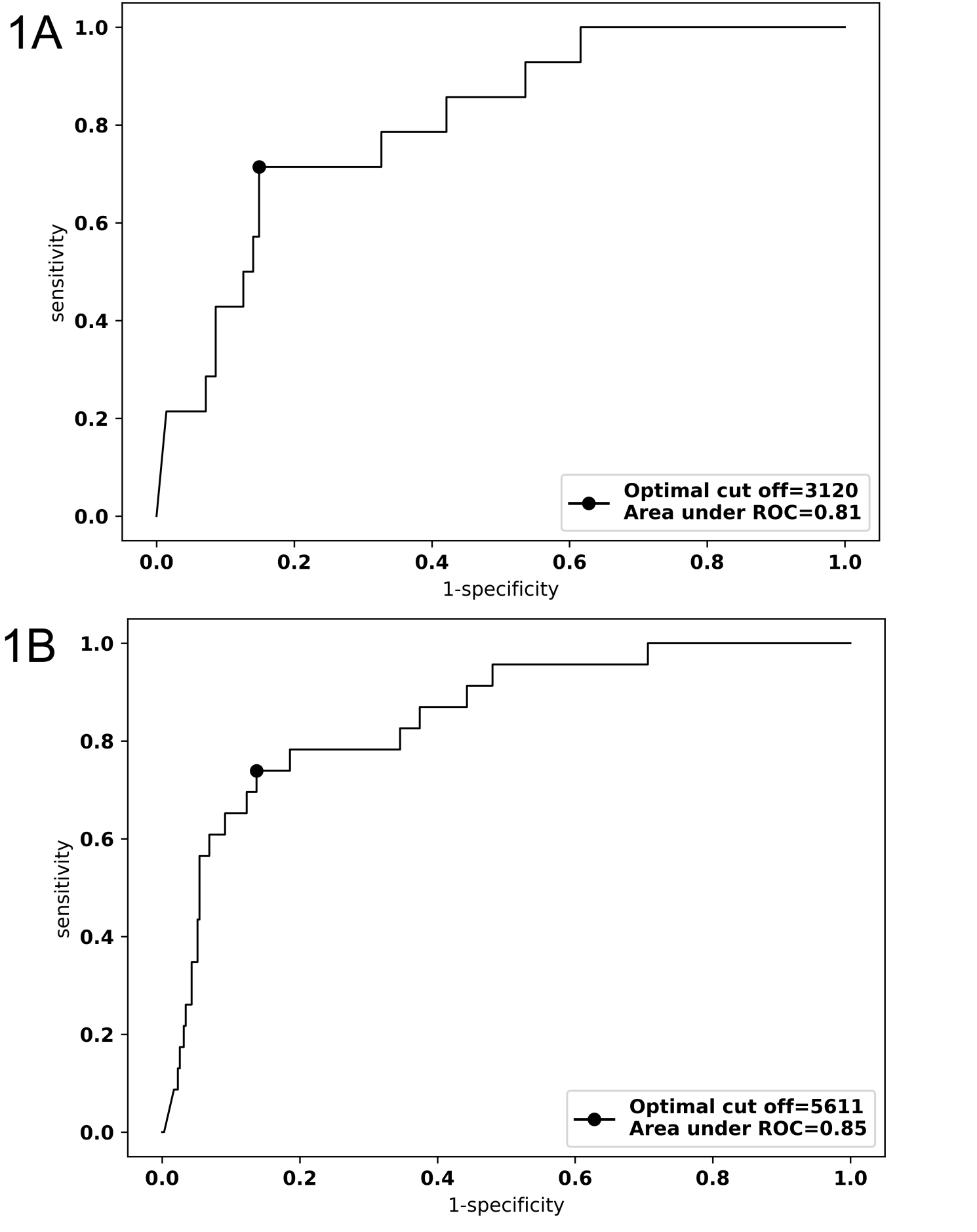

Supplement: Supplementary Materials — Supplementary Table 1: Descriptive analysis of patients with VTE upon admission or during hospitalization. Supplementary Figure 1: ROC curve of D-dimers in diagnosis of VTE among patients with VTE upon admission (1A), or during hospitalization (1B). [file 9126148.f1.docx]
